# Prediction of 90-Day Mortality among Sepsis Patients Based on a Nomogram Integrating Diverse Clinical Indices

**DOI:** 10.1155/2021/1023513

**Published:** 2021-10-20

**Authors:** Qingbo Zeng, Longping He, Nianqing Zhang, Qingwei Lin, Lincui Zhong, Jingchun Song

**Affiliations:** ^1^Intensive Care Unit, The 908th Hospital of Chinese PLA Logistical Support Force, Nanchang, China; ^2^Intensive Care Unit, Nanchang Hongdu Hospital of Traditional Chinese Medicine, Nanchang, China

## Abstract

**Background:**

Sepsis is prevalent among intensive care units and is a frequent cause of death. Several studies have identified individual risk factors or potential predictors of sepsis-associated mortality, without defining an integrated predictive model. The present work was aimed at defining a nomogram for reliably predicting mortality.

**Methods:**

We carried out a retrospective, single-center study based on 231 patients with sepsis who were admitted to our intensive care unit between May 2018 and October 2020. Patients were randomly split into training and validation cohorts. In the training cohort, multivariate logistic regression and a stepwise algorithm were performed to identify risk factors, which were then integrated into a predictive nomogram. Nomogram performance was assessed against the training and validation cohorts based on the area under receiver operating characteristic curves (AUC), calibration plots, and decision curve analysis.

**Results:**

Among the 161 patients in the training cohort and 70 patients in the validation cohort, 90-day mortality was 31.6%. Older age and higher values for the international normalized ratio, lactate level, and thrombomodulin level were associated with greater risk of 90-day mortality. The nomogram showed an AUC of 0.810 (95% CI 0.739 to 0.881) in the training cohort and 0.813 (95% CI 0.708 to 0.917) in the validation cohort. The nomogram also performed well based on the calibration curve and decision curve analysis.

**Conclusion:**

This nomogram may help identify sepsis patients at elevated risk of 90-day mortality, which may help clinicians allocate resources appropriately to improve patient outcomes.

## 1. Introduction

Sepsis is life-threatening organ dysfunction initiated by the body's overwhelming response to infection [1]. Although significant advances have been made in intensive care and supportive technology to treat sepsis, it remains associated with high morbidity and mortality. The global incidence rate is around 437 per 100 000 person-years, and approximately 17% of sepsis cases die in hospital [2]. These figures are even higher in China, where up to 20% of patients in intensive care units have sepsis [3].

The pathogenesis of sepsis is complex and involves coagulation disorder, inflammation imbalance, immune dysfunction, and mitochondrial and endothelial damage [4]. Better understanding of the disease's pathophysiology and identification of reliable predictors of short-term mortality are critical for guiding interventions and improving prognosis.

Several studies have analyzed risk factors for mortality in patients with sepsis [5–9], but most have focused on biomarkers related to inflammation or the function of certain organs. For such a complex disease, prediction algorithms may need to take a range of biomarkers into account. Therefore, the main objective of the present study was to consider a diversity of potential clinicodemographic factors for constructing a nomogram to predict 90-day mortality in sepsis.

## 2. Materials and Methods

### 2.1. Patient Selection and Data Collection

**Figure 1:**
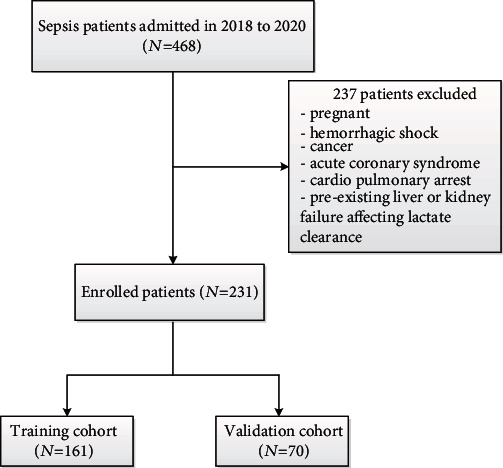
Flowchart of patients excluded by each criterion.

This retrospective study examined electronic medical record from a consecutive sample of 231 patients who had been diagnosed with sepsis and had been admitted to the intensive care unit at the 908^th^ People's Liberation Army Hospital (Nanchang, China) between May 2018 and October 2020. A flowchart of patients excluded by each criterion is shown in Figure 1. To be enrolled in the study, patients had to be older than 17 years and diagnosed with sepsis according to the Third International Consensus Definition for Sepsis (“Sepsis-3”) [10]: infection had to be confirmed through culture tests and the Sequential Organ Failure Assessment (SOFA) score had to be at least 2 [4]. Patients were excluded if they were pregnant or had a history of hemorrhagic shock, cancer, acute coronary syndrome, or cardiopulmonary arrest. This study was approved by the Ethics Committee of the 908^th^ People's Liberation Army Hospital with a waiver of informed consent. Baseline demographic data (age, sex) were collected, as were data on the site of infection, comorbidities, 90-day mortality, and severity of illness, based on the Acute Physiology and Chronic Health Evaluation II (APACHE II) score [11] and the SOFA scores [12] on the first day of admission to the intensive care unit, as well as numerous laboratory and clinical variables which were obtained four hours after admission (see Table 1).

### 2.2. Statistical Analysis and Nomogram Construction

All statistical analyses were performed using R 4.0.1 (R Core Team, Vienna, Austria) and SPSS 25.0 (IBM, Chicago, IL, USA). Differences associated with a two-sided *P* < 0.05 were considered statistically significant. Data for continuous variables were presented as mean ± standard deviation or as median (interquartile range (IQR)). Differences between groups were assessed for significance using Student's *t*-test in the case of normally distributed data or using the Mann-Whitney test in the case of a skewed distribution. Data for categorical variables were expressed as counts and percentages, and differences were assessed using *χ*^2^ or Fisher's exact test. The variance inflation factor (VIF) was used to test collinearity between continuous variables, and an arithmetic square root of VIF ≤ 10 was regarded as noncollinearity. Patients were randomized into training and validation cohorts in a ratio of 2 : 1. Clinical variables in the training cohort were entered into multivariate logistic regression, and backward stepwise selection was applied using the likelihood ratio test and Akaike's information criterion as the stopping rule [13]. The regression results from the training cohort were used to define a nomogram to predict 90-day mortality. The same regression equations for the training cohort were also applied to the data for the validation cohort in order to verify the nomogram. Calibration curves, accompanied by the Hosmer-Lemeshow test, were used to evaluate the predictive model. Its discriminative ability was assessed based on the area under the receiver operating characteristic curve (AUC). For clinical usefulness, net benefit was examined against the training and validation cohorts using decision curve analysis (DCA).

## 3. Results

### 3.1. Baseline Characteristics of Patients with Sepsis

Among the 231 patients in the study, 61.9% were men, the median age was 70 years (range, 18 to 96 years), and 73 (31.6%) died within 90 days of follow-up. In both the training and validation cohorts, patients who survived for 90 days had significantly lower levels of many clinical variables than those who died (Table 1), including tissue plasminogen activator-inhibitor complex, thrombin-antithrombin complex, prothrombin time, international normalized ratio, activated partial thrombin time, thrombin time, fibrinogen degradation product, D-dimer, creatinine, lactate, heart rate, Sequential Organ Failure Assessment, and Acute Physiology and Chronic Health Evaluation II. Conversely, survivors showed significantly higher levels of platelet, hemoglobin, and arterial partial oxygen pressure.

### 3.2. Nomogram Construction

**Figure 2:**
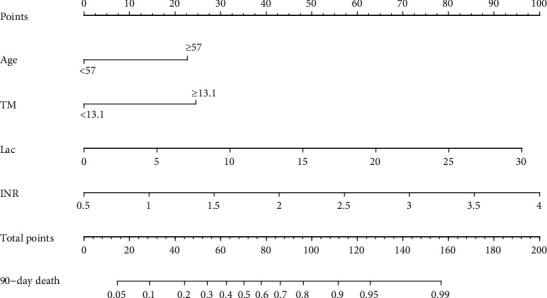
Nomogram for predicting 90-day mortality in patients with sepsis, based on data in the training cohort.

Multiple logistic regression identified age, international normalized ratio, lactate, and thrombomodulin as independent predictors of 90-day mortality (Table 2), which were then integrated into a predictive nomogram (Figure 2). The results of regression analysis were visualized. The clinician can give an individualized evaluation of the risk of 90-day mortality for patients undergoing sepsis according to the total points which were obtained by adding each score in the nomogram. This would facilitate precise risk assessment and better identification of 90-day mortality in the septic population.

### 3.3. Nomogram Validation

**Figure 3:**
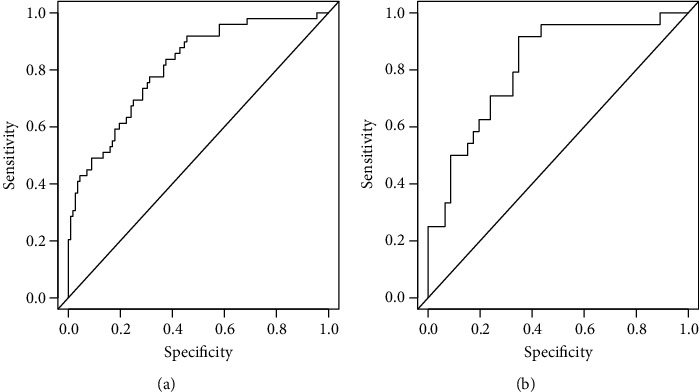
Receiver operating characteristic curves assessing the ability of the nomogram to predict 90-day mortality in (a) training and (b) validation cohorts.

The nomogram based on data in the training cohort gave an AUC of 0.810 (95% CI 0.739 to 0.881) for predicting 90-day mortality in that cohort (Figure 3(a)). Similarly, it gave an AUC of 0.813 (95% CI 0.708 to 0.917) for predicting 90-day mortality in the validation cohort (Figure 3(b)).

**Figure 4:**
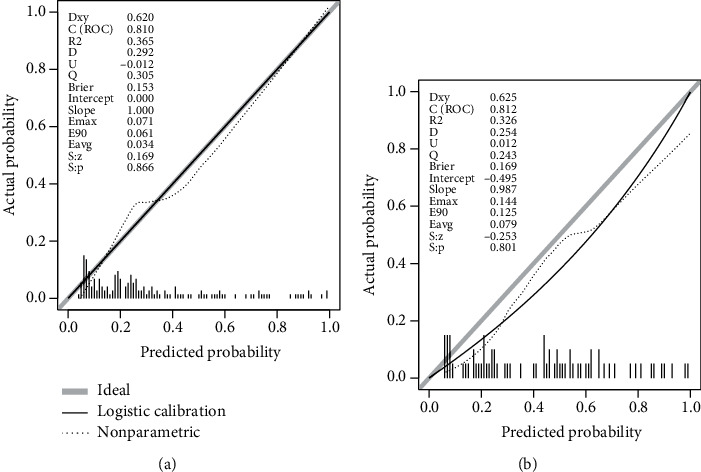
Calibration plot of predicted and observed probabilities of 90-day mortality in (a) training and (b) validation cohorts.

For both cohorts, the nomogram showed good agreement with actual 90-day mortality based on calibration curves (Figure 4), although the logistic calibration curve and nonparametric curve deviated slightly from the ideal line. The Hosmer-Lemeshow test gave a *P* = 0.866 in the training cohort while it gave a *P* = 0.801 in the validation cohort, suggesting no significant deviation from a perfect fit.

### 3.4. Potential Clinical Usefulness of the Nomogram

**Figure 5:**
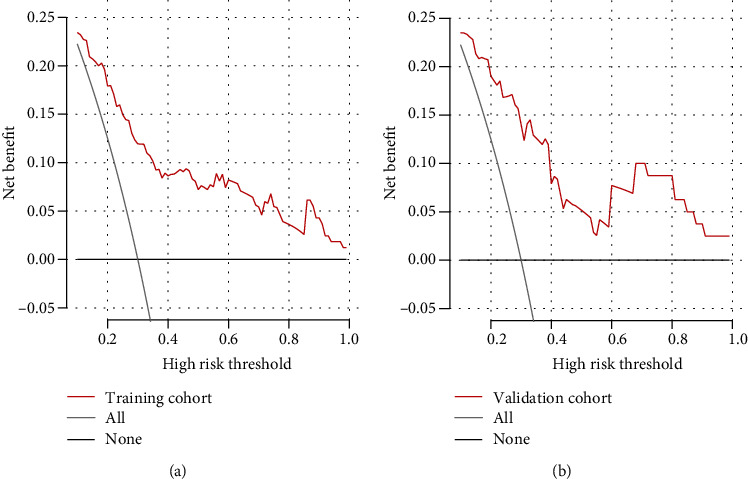
Decision curve analysis to assess the benefit of clinical intervention based on the predictive nomogram in (a) training and (b) validation cohorts.

DCA showed good clinical potential for the nomogram, based on the training cohort (Figure 5(a)) and validation cohort (Figure 5(b)). When the threshold probability is greater than 15%, using the nomogram can lead to lower mortality than treating either all or none of the patients.

## 4. Discussion

In this study, we defined a nomogram based on routinely measured clinical variables that may reliably predict 90-day mortality among patients with sepsis. While our nomogram should be verified with other patient populations, it establishes the feasibility of accurate mortality prediction using relatively simple clinical tests. While several studies have identified risk factors associated with 90-day mortality in sepsis, our work suggests that certain risk factors may be particularly relevant for screening patients for mortality risk.

The 90-day mortality in our retrospective cohort of Chinese patients was 31.6%, which was higher than that in previous studies [2, 3, 5]. Sepsis patients concluded in the present study had much higher APACHE II scores and had a longer follow-up (90-day mortality) than those in previous reports, which could explain these differences [14].

We found that the international normalized ratio was significantly higher among sepsis patients who died within 90 days of follow-up than among those who did not die, and it emerged as an independent predictor of 90-day mortality in multivariate analysis. Coagulopathy is frequently observed in sepsis [15], and it contributes to multiple organ dysfunction syndrome [16]. More severe coagulopathy has been linked to higher risk of mortality among patients with sepsis [17], and clinical parameters reflecting hemostasis can predict sepsis-related mortality [18–20]. Our results are consistent with this literature. Nevertheless, the international normalized ratio alone cannot accurately predict sepsis outcomes [5], which may be due to the need to take into account other independent predictors of mortality.

One of those predictors is lactate level, which was significantly higher among our patients who died within 90 days than among those who did not. Critically ill patients, particularly those with sepsis or septic shock, show elevated lactate [21], and the magnitude of the elevation correlates strongly and positively with sepsis severity and associated mortality [22–24]. Serum lactate levels are considered a marker of tissue hypoxia [19], and they have proven useful for guiding clinical treatment and predicting prognosis in various clinical contexts [25]. Our study supports the “Sepsis-3” recommendation that septic shock should be defined as persistence of serum lactate > 2 mmol/L [10].

Another risk factor for 90-day mortality that emerged as particularly important for prediction was elevated thrombomodulin level. Thrombomodulin, an integral endothelial cell membrane protein, is cleaved and released into the bloodstream during sepsis and septic shock [26, 27], leading to elevated levels of serum thrombomodulin in pediatric and adult sepsis patients [28, 29]. Endothelium is the primary site of damage in sepsis due to massive production of proinflammatory cytokines [6]. Elevated serum thrombomodulin level is associated with sepsis severity and risk of death [30]. Our study showed that endothelial cell injury justified by elevated TM activated the coagulation system, depleted coagulation factors characterized by prolonged PT to promote microthrombosis, and caused tissue hypoperfusion and increased lactate, especially obviously in elder patients with sepsis.

Our nomogram showed AUC values above 0.8 for the training and validation cohorts, suggesting good predictive ability. In addition, DCA suggested that treating our cohorts according to our nomogram's predictions could be superior to treating all or none of them. The calibration curve also suggested good fit. Nevertheless, our model was generated based on retrospective analysis of a relatively small sample from a single medical center, so it should be validated in other patient populations. It may be possible to further improve the model by a multicenter study with external validation.

## 5. Conclusions

We have developed a nomogram that may reliably predict 90-day mortality in patients with sepsis, based on age, international normalized ratio, lactate, and thrombomodulin. This may help clinicians identify patients at higher risk and modify clinical management and resource allocation accordingly.

## Figures and Tables

**Table 1:** Patient characteristics upon admission to the intensive care unit.

Characteristic	Training cohort			Validation cohort		
	Survivors (*n* = 112)	Died at 90 days (*n* = 49)	*P* value	Survivors (*n* = 46)	Died at 90 days (*n* = 24)	*P* value
Men	69 (61.6)	30 (61.2)	0.963	29 (63.0)	15 (62.5)	0.964
Age ≥ 57 yr	77 (68.8)	41 (83.7)	0.055	30 (65.2)	22 (91.7)	0.016
Comorbidity						
Diabetes	17 (15.2)	10 (20.4)	0.414	17 (37.0)	9 (37.5)	0.964
Hypertension	42 (37.5)	26 (53.1)	0.066	21 (45.7)	12 (50.0)	0.729
COPD	6 (5.4)	6 (12.2)	0.126	8 (17.4)	6 (25.0)	0.450
CKD	10 (8.9)	6 (12.2)	0.518	2 (4.3)	3 (12.5)	0.209
Source of infection						
Pulmonary	71 (63.4)	33 (67.3)	0.629	27 (58.7)	19 (71.2)	0.087
Urinary tract	8 (7.1)	1 (2.0)	0.195	5 (10.9)	0 (0)	0.094
Abdominal	27 (24.1)	14 (28.6)	0.550	13 (28.3)	3 (12.5)	0.136
Skin	6 (5.4)	2 (4.1)	0.732	2 (4.3)	1 (4.2)	0.972
TM ≥ 13.1 TU/mL	48 (42.9)	32 (65.3)	0.010	17 (37.0)	17 (70.8)	0.007
TAT (ng/mL)	8.2 (4.6-18.0)	17.2 (5.7-46.8)	0.002	8.7 (5.6-17.0)	13.4 (6.2-30.9)	0.162
PIC (*μ*g/mL)	1.16 (0.62-2.16)	1.04 (0.57-2.28)	0.742	1.10 (0.75-1.48)	1.43 (0.69-2.83)	0.421
t-PAIC (ng/mL)	12.2 (7.6-24.1)	21.7 (11.3-41.7)	0.003	14.2 (9.4-23.9)	21.3 (13.8-47.1)	0.020
PT (s)	14.2 (12.7-16.2)	16.4 (14.0-21.4)	0.000	13.7 (13-15.3)	15 (13.6-19.6)	0.008
INR	1.2 (1.1-1.3)	1.4 (1.2-1.8)	0.000	1.14 (1.08-1.27)	1.25 (1.13-1.60)	0.008
APTT (s)	31.6 (26.6-38.4)	37.4 (32.0-47.7)	0.000	31.4 (26.7-40.5)	33.9 (29.2-48.7)	0.087
FIB (g/L)	2.9 ± 1.09	2.6 ± 1.2	0.143	2.9 ± 0.9	2.7 ± 1.3	0.308
TT (s)	15.8 (14.5-17.3)	17.2 (14.8-18.7)	0.003	15.5 (14.0-17.4)	16.8 (14.9-19.4)	0.070
FDP (*μ*g/L)	8.69 (3.67-18.92)	14.45 (4.53-38.00)	0.030	7.56 (4.51-13.12)	11.37 (6.99-27.95)	0.033
D-dimer (*μ*g/L)	2.59 (1.03-5.97)	4.91 (1.65-11.00)	0.016	2.19 (0.87-4.53)	3.19 (2.54-7.76)	0.015
Platelets (×10^9^/L)	179 ± 90	138 ± 94	0.010	182 ± 108	209 ± 128	0.358
Hemoglobin (g/L)	111 ± 29	100 ± 31	0.038	109 ± 31	104 ± 31	0.525
ALT (U/L)	31.9 (12.9-73.5)	21.5 (13.3-116.8)	0.597	27.3 (13.3-58.7)	29.6 (11.1-64.2)	0.921
AST (U/L)	43.0 (23.3-84.3)	42.1 (26.4-131.2)	0.483	33.2 (19.8-72.5)	30.3 (19.1-76.7)	0.843
TBil (*μ*mol/L)	13.5 (7.9-22.5)	13.9 (7.4-32.5)	0.514	14.5 (6.8-23.4)	17.6 (10.9-28.1)	0.192
Cr (*μ*mol/mL)	92.6 (62.3-163.8)	136 (76.5-241.4)	0.017	70.3 (54.5-132.5)	113.4 (78.3-150.0)	0.056
RBG (mmol/L)	7.3 (6.2-9.3)	6.8 (5.5-9.1)	0.142	7.6 (6.7-9.6)	8.8 (7.1-10.5)	0.239
Body temp (°C)	36.7 (36.5-37.5)	36.6 (36.3-37.3)	0.350	36.7 (36.2-37.3)	36.4 (36.0-36.8)	0.176
Heart rate (min^−1^)	96 ± 20	106 ± 25	0.013	98 ± 26	107 ± 26	0.179
MAP (mmHg)	90 ± 17	88 ± 22	0.547	91 ± 17	87 ± 18	0.352
SOFA score	7 (5-10)	9 (7-15)	0.000	7 (5-10)	9 (6-13)	0.065
APACHE II score	21 ± 6	24 ± 6	0.008	22 ± 7	27 ± 7	0.004
PH	7.41 (7.35-7.45)	7.38 (7.29-7.50)	0.133	7.42 (7.34-7.49)	7.29 (7.19-7.43)	0.006
PaCO_2_ (mmHg)	36 (31-42)	34 (29-40)	0.149	34 (28-41)	39 (32-46)	0.040
PaO_2_ (mmHg)	110 (81-157)	93 (64-140)	0.017	112 (80-167)	97.1 (64-152)	0.366
Lac (mmol/L)	1.7 (1-3.2)	3.2 (1.5-6.6)	0.000	2.0 (1.2-3.3)	3.8 (1.8-9.5)	0.010

Values are *n* (%), mean ± SD, or median (interquartile range). Abbreviations: COPD: chronic obstructive pulmonary disease; CKD: chronic kidney disease; TM: thrombomodulin; TAT: thrombin-antithrombin complex; PIC: *α*2-plasmininhibitor-plasmin complex; tPAIC: tissue plasminogen activator-inhibitor complex; PLT: platelet; HB: hemoglobin; PT: prothrombin time; APTT: activated partial thrombin time; FIB: fibrinogen; INR: international normalized ratio; TT: thrombin time; FDP: fibrinogen degradation product; ALT: alanine transaminase; AST: aspartate transaminase; TBil: total bilirubin; MAP: mean arterial pressure; SOFA: Sequential Organ Failure Assessment; APACHE II: Acute Physiology and Chronic Health Evaluation II; pH: potential of hydrogen; PaO_2_: arterial partial oxygen pressure; PaCO_2_: arterial partial pressure of carbon dioxide; Lac: lactate; RBG: random blood glucose.

**Table 2:** Multivariate logistic regression of data from the training cohort to identify factors independently associated with 90-day mortality.

Variable	Odds ratio	95% confidence interval	*P* value
Age (≥57 vs. <57 y)	1.20	0.36-2.04	0.005
TM (≥13.1 vs. <13.1 TU/mL)	1.30	0.39-2.21	0.005
INR	1.52	0.23-2.80	0.021
Lac (mmol/L)	0.17	0.04-0.29	0.008

INR: international normalized ratio; TM: thrombomodulin; Lac: lactate.

## Data Availability

The raw data supporting the conclusions of this article will be made available by the authors, without undue reservation.
